# Differential sensitivity of impedance plethysmography and photoplethysmography sensors to temperature-induced peripheral vasoconstriction

**DOI:** 10.1038/s41598-026-36563-6

**Published:** 2026-01-31

**Authors:** Seobin Jung, Seamus Thomson, Alexandros Pantelopoulos, Lindsey Sunden, Peter Richards, Shwetak Patel, Sam Sheng

**Affiliations:** Google, 1600 Amphitheatre Pkwy, Mountain View, CA 94043 USA

**Keywords:** Impedance plethysmography (IPG), Photoplethysmography (PPG), Vasoconstriction, Blood flow, Hemodynamics, Blood pressure, Heart rate, Wearables, Sensors, Peripheral, Skin temperature, Physiology, Biomedical engineering

## Abstract

Impedance plethysmography (IPG) and photoplethysmography (PPG) are non-invasive techniques for measuring blood volume changes. This study investigated the differential responses of IPG and PPG to temperature-mediated vasoconstriction induced by localized cooling. Twenty-one participants underwent control and treatment conditions, with fake or real ice cubes applied to the forearm. Blood pressure remained stable, while heart rate decreased. PPG signal amplitude significantly decreased with cooling (p_adj_ = 0.004), indicating sensitivity to superficial blood flow changes. In contrast, IPG signal amplitude remained stable (p_adj_ = 1.0). No statistically significant differences were observed in timing-derived metrics. These findings suggest IPG is less sensitive to superficial changes in blood flow than PPG, and may be more suitable for monitoring deeper blood flow. This study provides insights into the distinct sensitivities of IPG and PPG, with implications for wearable device development and cardiovascular monitoring.

**Supplementary Information:**

The online version contains supplementary material available at 10.1038/s41598-026-36563-6.

## Introduction

**Fig. 1:**
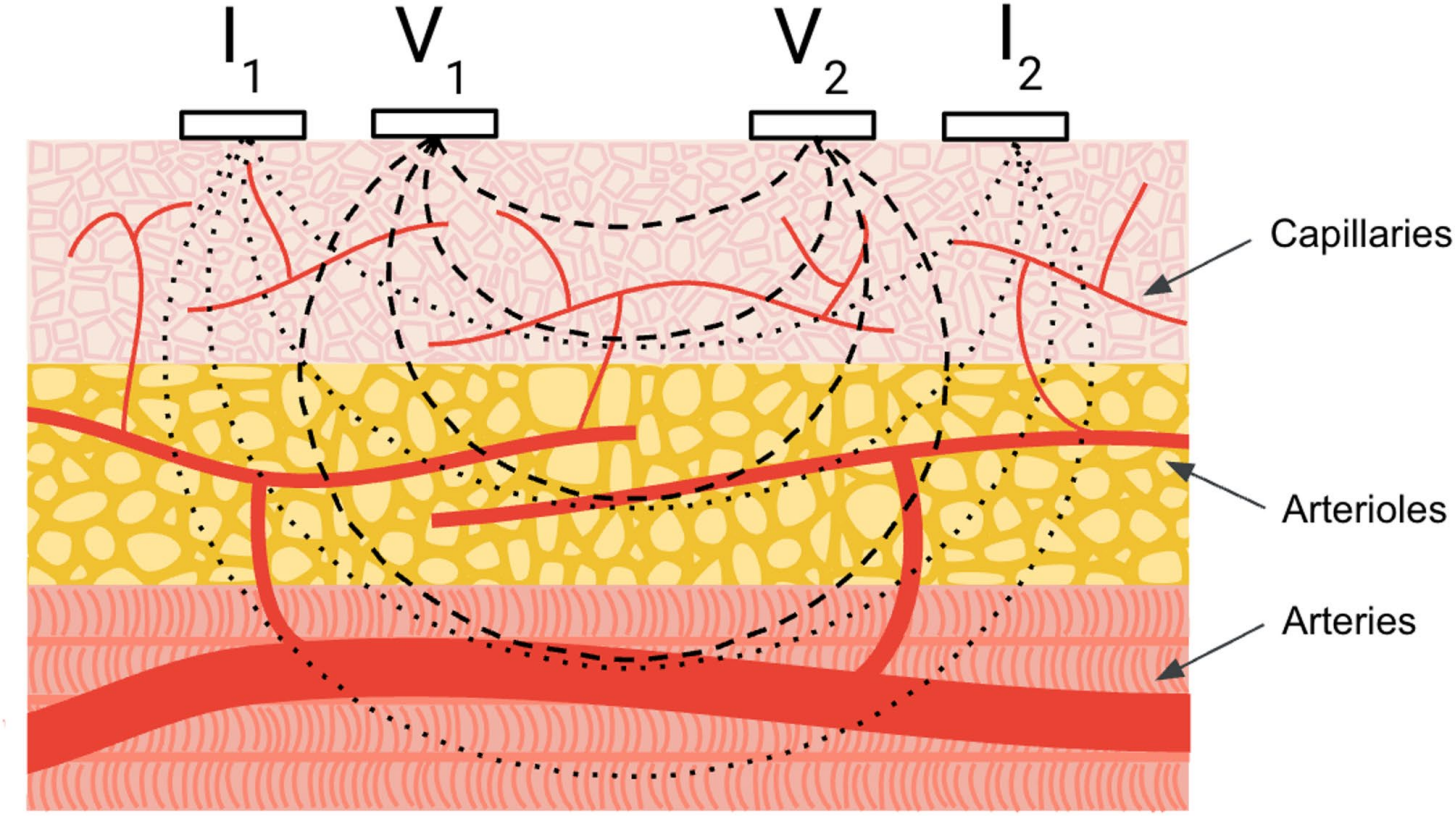
Illustrative cross-sectional representation of four-electrode impedance plethysmography setup for measuring blood volume variations with electrodes positioned on top of the skin (not to scale). Current injection electrodes (I_1_ and I_2_) establish an electric field within the stratified tissues. Voltage sensing electrodes (V_1_ and V_2_) measure the resulting impedance, which is modulated by pulsatile blood flow in the underlying vascular structures. Dotted lines approximate the current flow and dashed lines conceptually indicate the region influencing the impedance measurement.

 Impedance plethysmography (IPG) is a non-invasive technique for measuring blood flow in the periphery^1^. Initially developed for monitoring vascular conditions such as deep vein thrombosis and peripheral arterial disease^2^, IPG has more recently been employed in wearable sensor research^3^. The sensing mechanism of IPG involves applying an alternating current between two outer electrodes, and measuring the resulting voltage potentials with two inner electrodes, producing time-series impedance data (Fig. 1). This is the same principle as impedance cardiography (ICG) which uses a tetrapolar electrode on the central part of one’s body to monitor mechanical heart health^4^. While traditionally confined to clinical settings for assessing vascular health, advancements in wearable sensor technology have broadened the potential applications of IPG. For example, recent studies report using IPG for the passive monitoring of pulsatile waveforms in the periphery towards the development of cuffless blood pressure estimation models^3,5,6^ or respiratory sensing^7^. Despite these advancements, a comprehensive understanding of IPG’s sensitivity to blood flow - particularly in smaller, superficial blood vessels such as capillaries - and sensing depth remains an active area of research.

Photoplethysmography (PPG) is another non-invasive optical technique that measures pulsatile signal, frequently used in wearable devices for deriving heart rate and other cardiovascular features^8^. It is well known that PPG is highly sensitive to superficial blood flow^9^. While the precise underlying physiological origins of the PPG signal are still debated, with leading models including red blood cell dynamics, blood volume changes, and capillary mechanical movements, there is a general consensus that it primarily reflects microvascular perfusion^10–12^. The extensive study of PPG has led to a comprehensive understanding of its signal characteristics, such as penetration depth and its corresponding factors (e.g., wavelength, intensity, source-detector distance, etc.)^13^. Conversely, research on the fundamental origin of the IPG signal and its signal penetration depth is considerably more limited^14^.

One empirical approach to evaluate the physiological origin and penetration depth of biomedical signals involves leveraging phenomena unique to specific anatomical layers, such as peripheral vasoconstriction. Temperature-mediated vasoconstriction, a well-documented physiological phenomenon, is known to reduce superficial blood flow^15^. Specifically, exposure to cold stress has been observed to cause a reduction in PPG peak amplitudes^16^. This leads to a critical, unanswered question: how does temperature-mediated vasoconstriction affect IPG signal? Does it, like PPG, manifest as a change in signal amplitude? By positioning a PPG sensor between two IPG sensing electrodes on top of the radial artery and inducing a decrease in skin temperature, we can simultaneously observe the effects of vasoconstriction on both IPG and PPG signals.

This study aims to investigate the distinct effects of reduced superficial blood flow on IPG signals by comparing them to simultaneously-acquired PPG signals, an established indicator of superficial microvascular perfusion. By inducing localized cooling-induced vasoconstriction, we seek to elucidate how changes in superficial blood flow manifest in both IPG and PPG waveforms. Additionally, the temporal characteristics of these signals - such as pulse arrival time (PAT) and the relative timing between different plethysmographic signals - are examined to see if they offer additional insights into vascular stiffness, blood pressure dynamics, and the depth of signal penetration^8,17^. This investigation will contribute to a more nuanced understanding of IPG’s physiological origins and its sensitivity to microvascular changes, informing its potential for enhanced cardiovascular monitoring and broader integration into wearable devices.

## Methodology

### Participants

Participants were recruited from a pool of Google full-time employees aged 18 years or older, located within the United States of America, capable of giving informed consent, wearing test devices for extended periods, and participating in test protocols while wearing validated research devices. Participants were excluded if they were unwilling or unable to provide informed consent, wear test devices for extended periods of time, unable to participate in test protocols while wearing validated research devices, or have presence of potential identifiers or markings on the wrist region where thermal images are taken.

### Instrumentation and signal processing

**Fig. 2:**
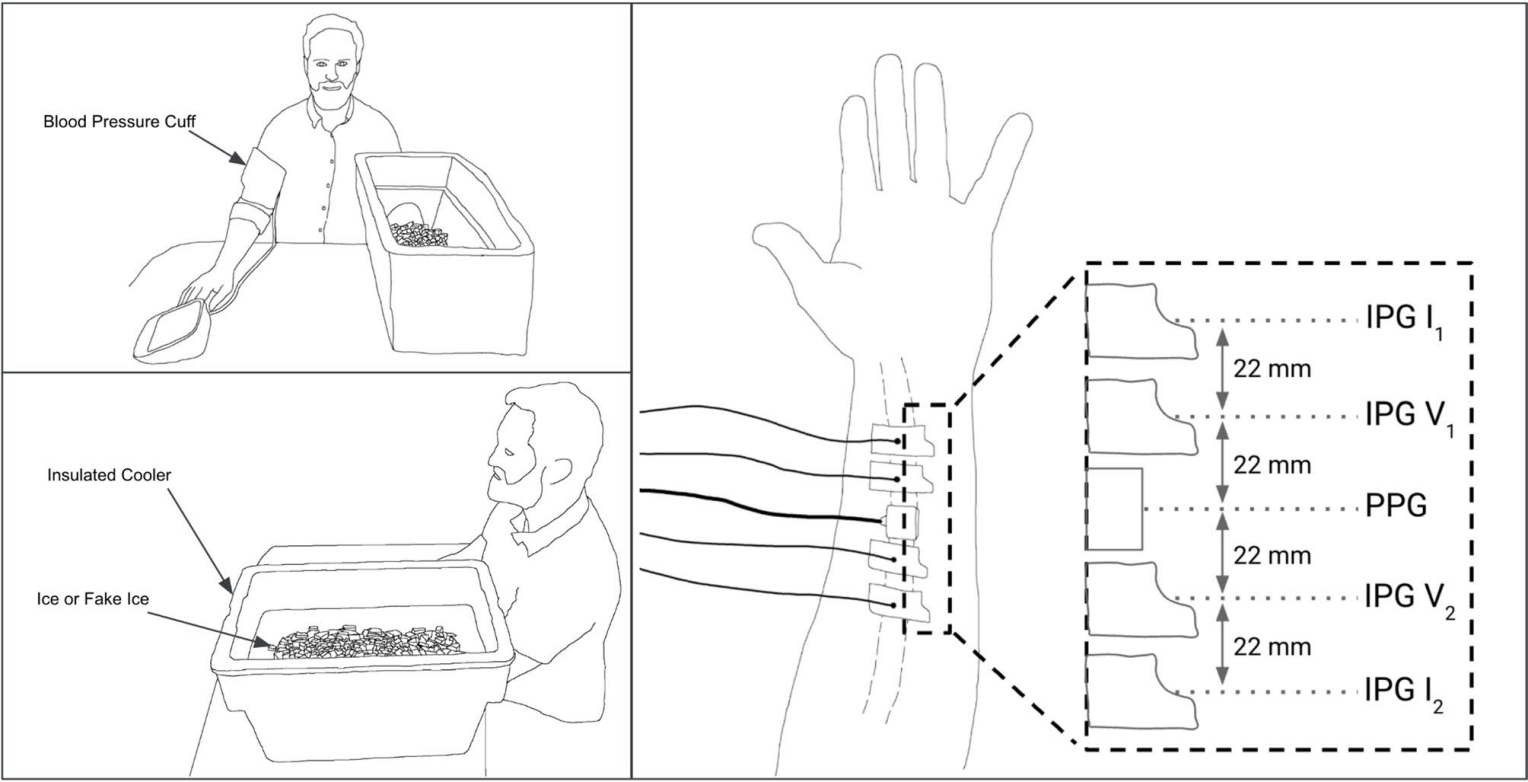
Experimental setup and sensor configurations. The left two panels depict the overall setup including the participant, insulated cooler used for the cold stimulus, and the position for the blood pressure measurement. The right panel shows the placement of four IPG electrodes and PPG module along the forearm targeting the approximate location of the radial artery.

An infrared thermal camera (FLIR E8) served the dual purpose of identifying the radial artery and recording skin temperature. IPG signals were acquired using 3 M 2760-5 gel electrodes positioned with a custom alignment guide over the radial artery region as shown in Fig. 2 right panel. A PPG sensor was placed between the IPG electrodes and secured on each participant with an elastic band to ensure a snug fit and consistent positioning. An automated blood pressure cuff (Omron BP7450) was placed on the opposite arm. Electrocardiogram (ECG) signals were recorded using gel electrodes configured for lead I (left shoulder, right shoulder, and right leg ankle). Physiological signals were acquired using a Biopac MP36R system with dedicated modules for IPG (Biopac SS31L; 100 kHz excitation frequency, 400 µA_rms_ continuous excitation current), PPG (Biopac SS4LA; 860 nm ± 60 nm infrared, 5 mA continuous light emitting diode (LED) current drive, 3.81 mm LED-to-photodiode spacing), and ECG (Biopac SS2LB).

**Fig. 3:**
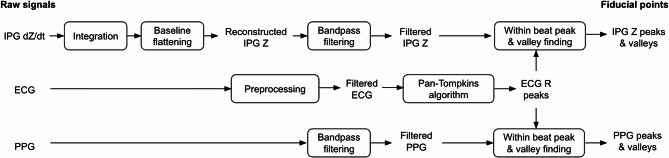
Illustration of the signal processing and peak/valley identification pipeline used for ECG, IPG and PPG.

**Fig. 4:**
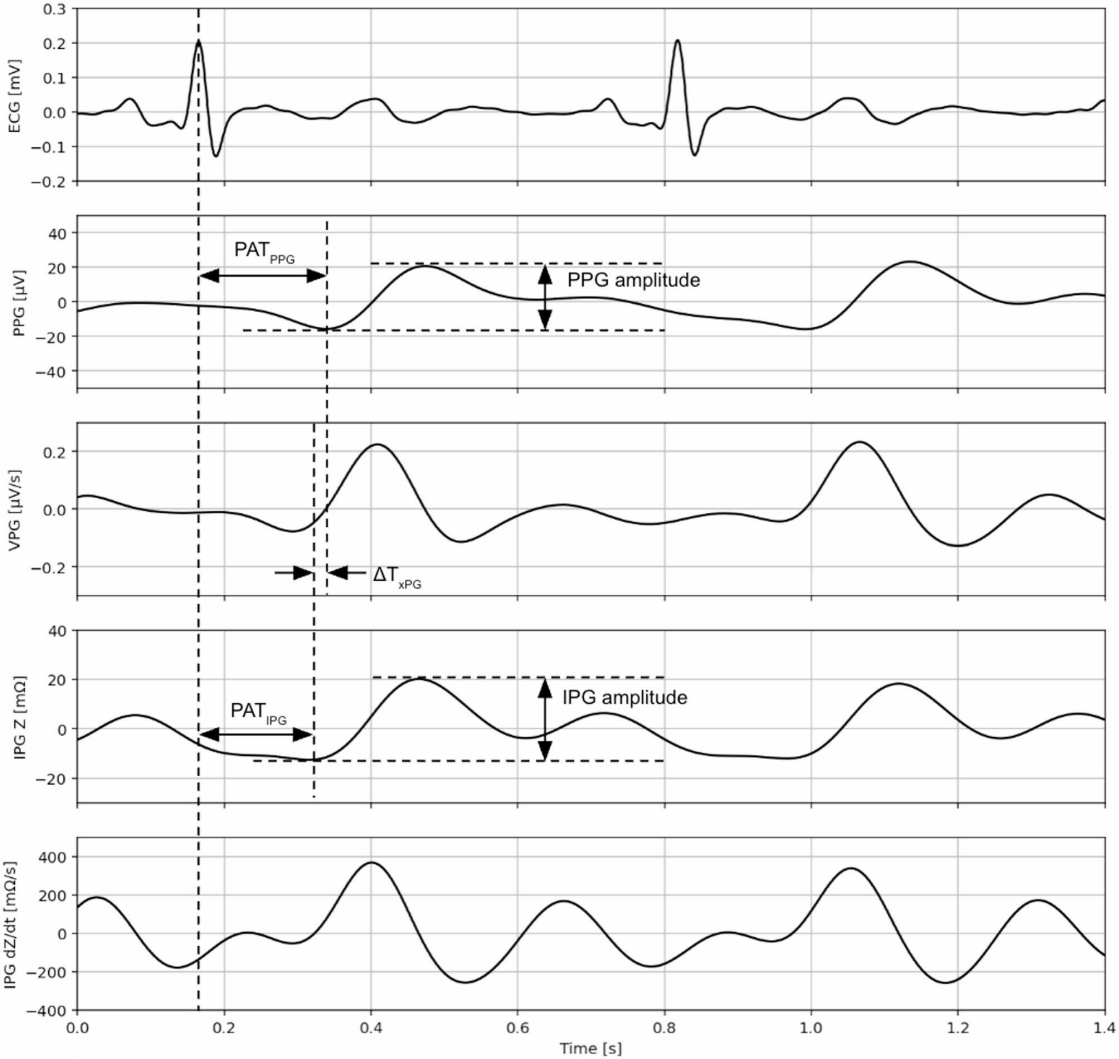
Illustration of feature extraction from simultaneously acquired cardiovascular signals. The traces display ECG, PPG, velocity plethysmography (VPG, first time derivative of PPG), IPG Z, and IPG dZ/dt. Indicated parameters include peak amplitudes for PPG and IPG, and timing features such as pulse arrival time for PPG and IPG referenced to the preceding ECG R peak, and the time interval between PPG valley and IPG Z valley (ΔT_xPG_).

Blood pressure, temperature readings, and sensor data (IPG, PPG, and ECG) were analyzed using Python. Biopac sensor data were recorded at a sampling rate of 2 kHz with time synchronization. The signal processing pipeline for Biopac sensor data is summarized in Fig. 3. For ECG, R peaks were found using the Pan-Tompkins algorithm following preprocessing involving powerline noise removal via notch filters and denoising with a bandpass filter (5 to 35 Hz, Butterworth, 14th order in total, applied forward and backward). Similarly, prior to peak detection, PPG and IPG signals were bandpass filtered (0.5 to 12 Hz, Chebyshev type II, 4th order in total, applied forward and backward) with a minimized phase shift. The Biopac IPG Z channel often exhibiting saturation (a characteristic attributed to its hardware-based filtering in the IPG module) or a relatively large quantization step size (~ 3 mΩ), which necessitated additional processing to address this challenge. The IPG Z signal was reconstructed by first integrating its dZ/dt channel using the cumulative_trapezoid method from the Python package Scipy. This integrated Z signal then underwent baseline flattening using a moving average subtraction method with a window size of 2000 samples. The baseline detrending was deemed acceptable as this study primarily cares about pulsatile signal components, which were kept intact. Exemplary filtered waveform traces from a representative participant are shown in Fig. 4.

For statistical analysis, paired t-tests were performed using the Scipy Python package. Two-way repeated measures ANOVA tests were conducted using the Pingouin Python package. Bonferroni correction was applied for multiple comparisons (adjusted p-values, p_adj_).

### Procedure

This study employed a within-subject design wherein participants were sequentially exposed to both control and treatment conditions. The procedure was identical for both conditions, with the exception of the stimuli applied. In the treatment condition, real ice cubes were used to induce localized change in temperature and peripheral vasoconstriction. For the control condition, which happened before the treatment condition, fake ice cubes were applied to replicate potential physical disturbances to the sensors such as pressure without inducing a temperature change.

Participants were instructed to position their arm with the attached sensors into an insulated cooler placed on a table, ensuring the arm remained parallel to the table surface with the ventral wrist facing upwards (Fig. 2 left panel). Throughout the data collection, participants were instructed to be silent and maintain physical stillness. Signal quality was visually assessed in real-time during data acquisition. If the data exhibited unusual artifacts, high noise, or no clear pulsatile, proctors stopped the data collection and performed troubleshooting steps such as looking for potential connection errors, sensor misplacement, or unintentional movement.

**Fig. 5:**
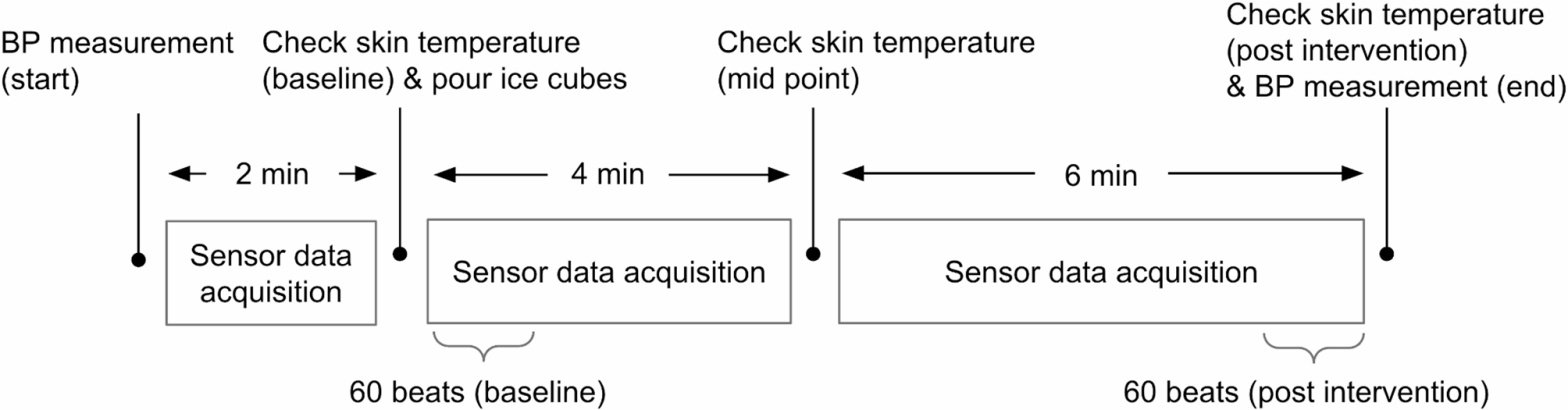
Schematic diagram of the experimental procedure and timeline. Key steps include baseline and post-intervention BP measurements, skin temperature checks, application of cold stimulus, and defined periods of sensor data acquisition. Analysis segments correspond to 60 heartbeats during the start of the baseline and end of the post-intervention phases. The experimental procedure was the same for both the control condition and the treatment condition except the ice cubes being fake vs. real. BP measurements were done at the start of the control and the end of the treatment condition.

Following an initial blood pressure measurement and the placement of a towel over the participant’s arm, IPG, PPG, and ECG signals were recorded for two minutes. Subsequently, skin temperature was measured at three distinct locations: proximally and distally adjacent to the outer IPG electrodes, and centrally adjacent to the PPG sensor. Approximately 1.6 L of either real or fake ice cubes, depending on the assigned condition, were then placed on the towel, covering the forearm with particular attention to the sensor area. After 4 min and then another 6 min later, the towel was briefly lifted to retake temperature measurements. Finally, signal acquisition was stopped and a final blood pressure reading was acquired. For analysis, “baseline” and “post intervention” periods were defined as the 60 beats immediately after ice placement and the final 60 beats of signal recording, respectively (Fig. 5).

### Ethical considerations

All participants provided informed consent prior to their participation in the study. The study protocol was approved by the WIRB-Copernicus Group (WCG) Institutional Review Board under the protocol number 20,243,288. All experiments were performed in accordance with relevant guidelines and regulations.

## Results

### Participant demographics

This study included 21 participants, with a mean age of 35.3 ± 6.6 years and a semi-balanced gender distribution (9 females, 12 males). Demographic information is presented in Table 1, including age, gender, height, weight, body mass index (BMI), self-assessed Monk Skin Tone Scale, and self-assessed Fitzpatrick Scale. For continuous variables, data are reported as mean ± standard deviation and range (minimum-maximum). Categorical variables are presented as counts and percentages for each category.

**Table 1:** Participant demographics.

Characteristic	Value
Age (years)	35.3 ± 6.6 (23–57)
Gender (Male/Female)	12 (57%)/9 (43%)
Weight (kg)	75.7 ± 17.4 (50.0–111.1)
Height (cm)	174.7 ± 9.2 (157.5–188.0)
BMI (kg/m^2^)	24.6 ± 4.2 (17.2–35.2)
Monk Skin Tone Scale	A: 0 (0%), B: 5 (23.8%), C: 2 (9.5%), D: 6 (28.6%), E: 4 (19%), F: 2 (9.5%), G: 2 (9.5%), H: 0 (0%), I: 0 (0%), J: 0 (0%)
Fitzpatrick Scale	I: 2 (9.5%), II: 4 (19%), III: 8 (38%), IV: 5 (24%), V: 2 (9.5%), VI: 0 (0%)

### Skin temperature and blood pressure analysis

**Fig. 6:**
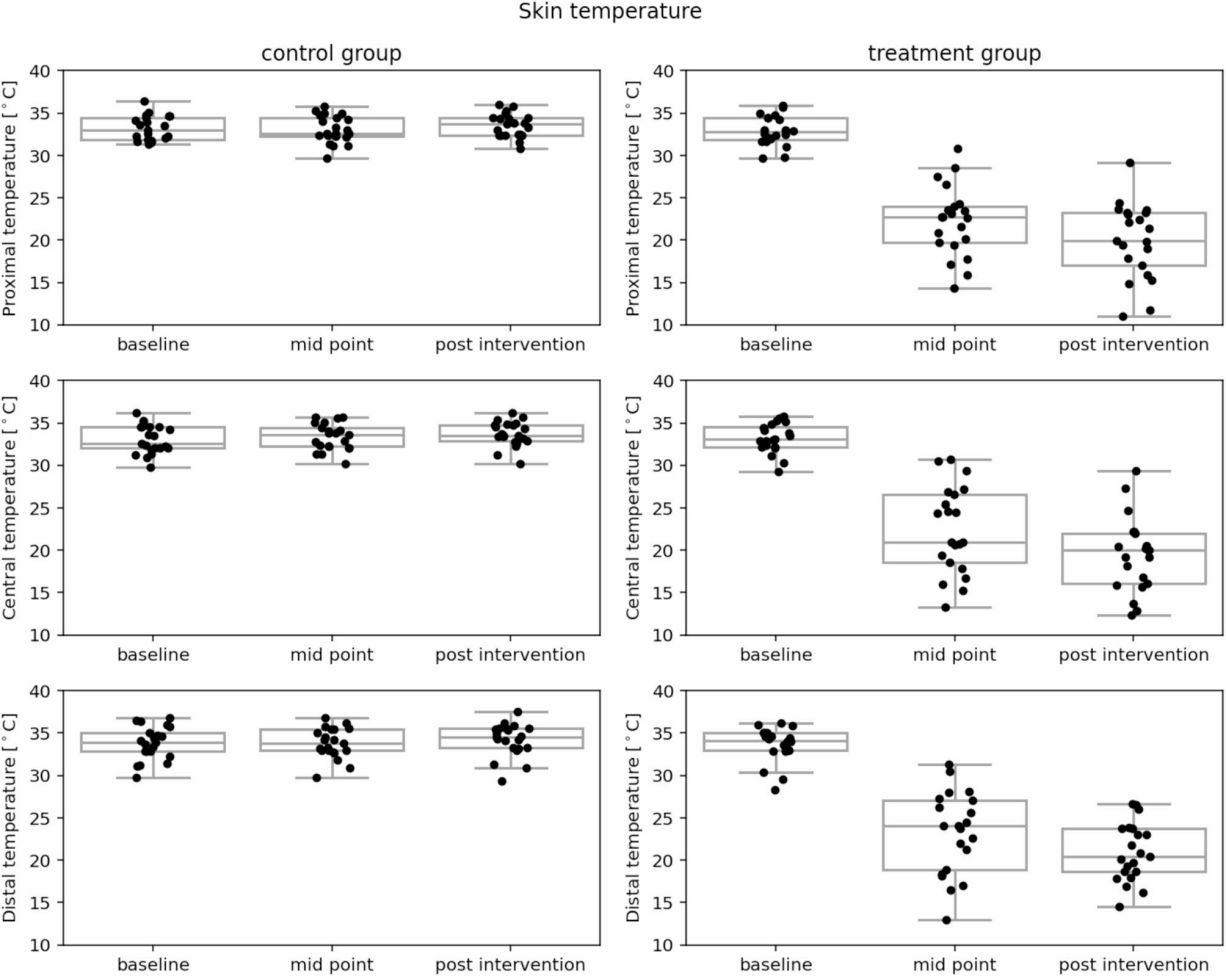
Comparison of proximal, central, and distal skin temperatures [unit: ℃] between the control and treatment groups. Measurements were taken at baseline, mid point of the intervention, and post intervention. Individual points show data for each participant and box plots indicate the collective distributions.

The ice cube intervention decreased skin temperature in the treatment group by an average of 13.2 °C across the three measurement locations. In contrast, the control group experienced a slight temperature increase of 0.5 °C, likely due to the insulating effect of the towel (Fig. 6). Paired t-tests revealed a statistically significant difference (p_adj_ < 0.001) in skin temperature between the treatment and control groups across the three measurement sites (Table 4, Appendix).

**Fig. 7:**
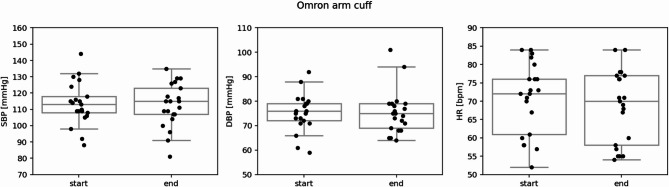
Omron arm cuff measurements of SBP [unit: mmHg], DPB [unit: mmHg], and HR [unit: bpm] at the start and end of the experimental protocol. Individual points show data for each participant and box plots indicate the collective distributions.

Blood pressure (systolic and diastolic) did not significantly change between the start and end of the measurement period. Heart rate, however, did significantly decrease (*p* < 0.05) by a mean of 2.1 beats per minute (Fig. 7; Table 5, Appendix).

### Signal amplitude analysis

**Fig. 8:**
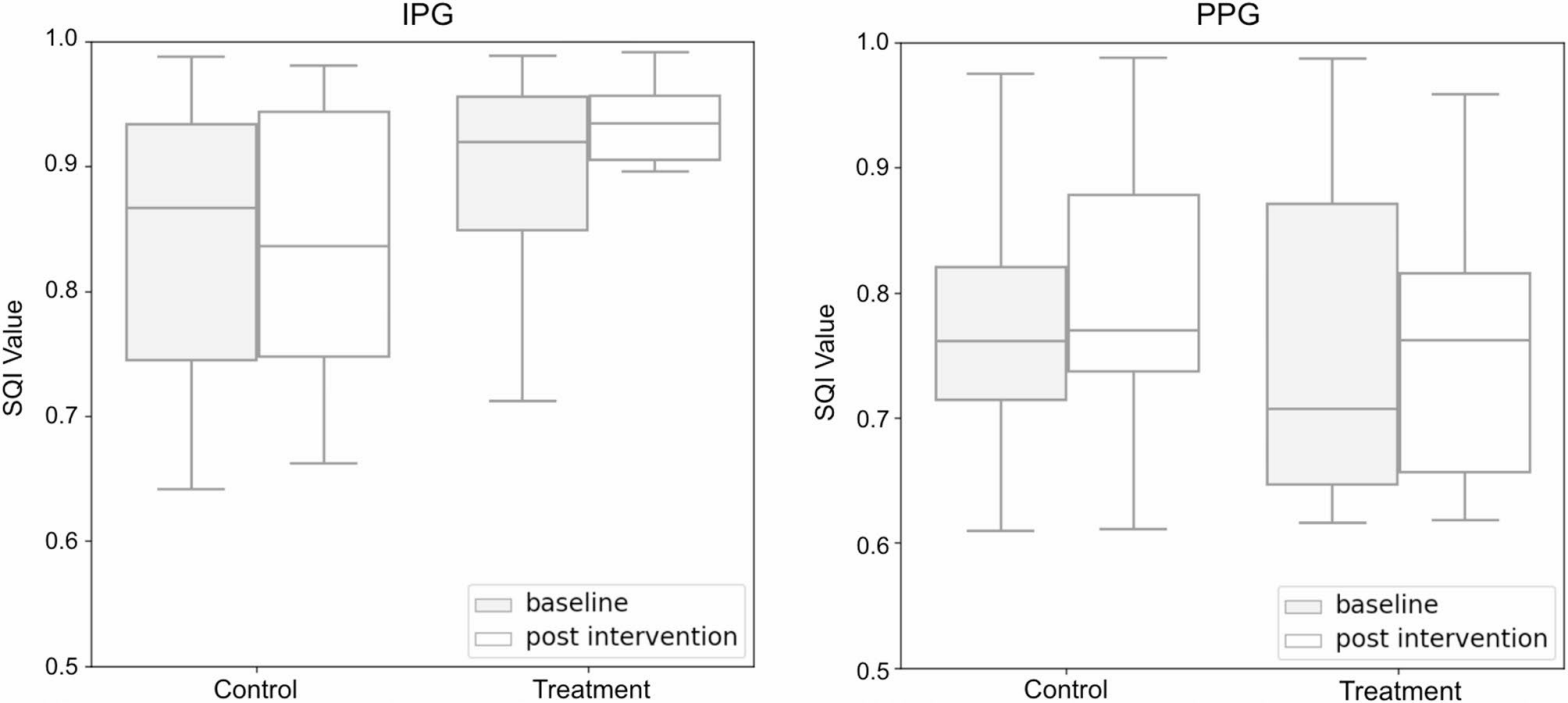
Distribution of SQI values for IPG and PPG beats across control and treatment groups. 60 heartbeats were analyzed for baseline and post intervention respectively as shown in Fig. 5.

**Fig. 9:**
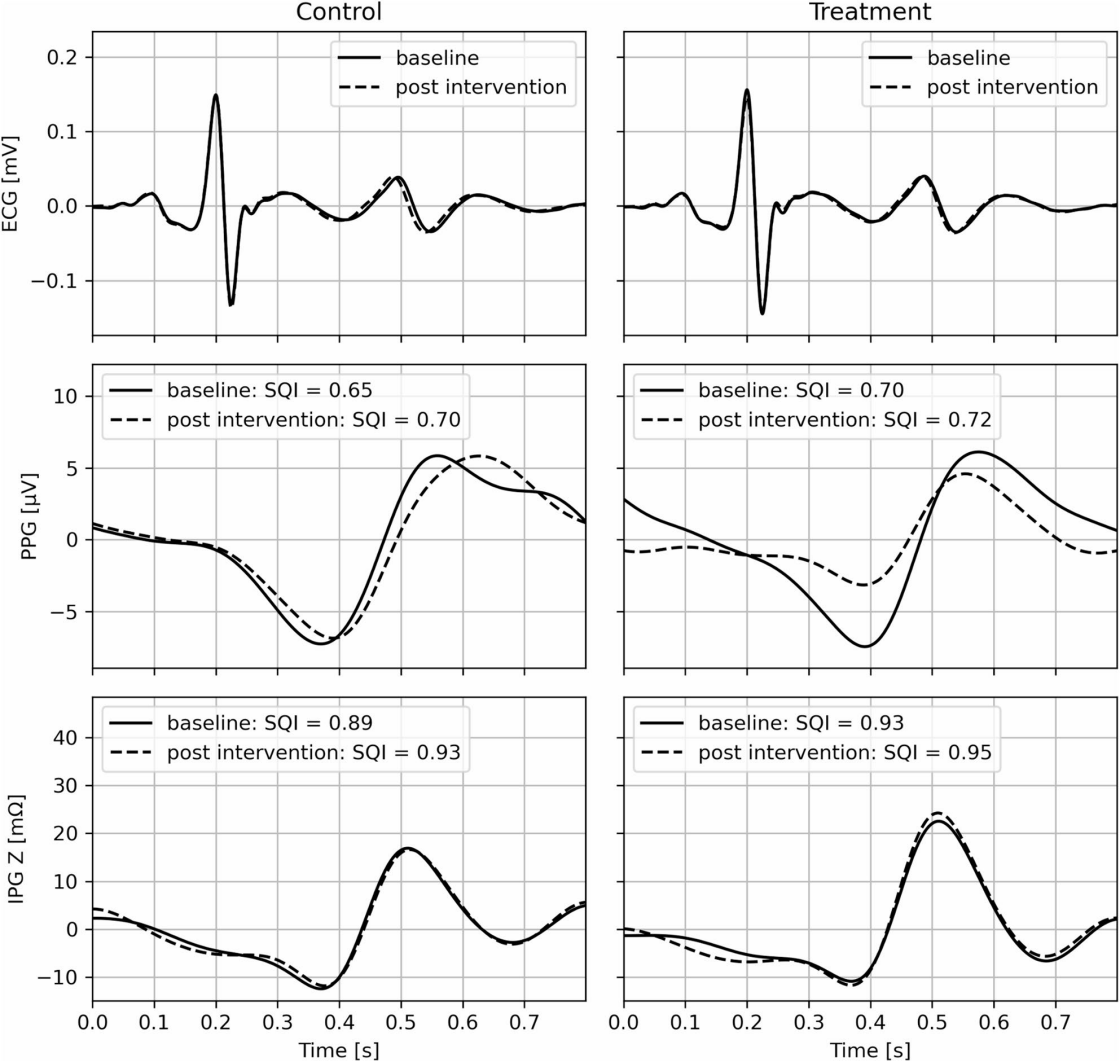
An exemplary aggregated waveform of ECG, PPG, and IPG Z across measurement phases (solid line: baseline, dashed line: post intervention) and procedural groups (left: control, right: treatment) from one participant. SQI values are labeled.

Both PPG and IPG used the same peak finding approach which consisted of being guided by ECG R peaks to scope one beat interval and finding minima (for valley) and maxima (for peak) within the beat interval. PPG and IPG amplitude features using this method are shown in Fig. 4. For each data collection phase (baseline and post intervention), peak-to-peak amplitudes were calculated from 60 consecutive beats (Fig. 5). Table 2 reports mean ± standard deviation (SD) of PPG and IPG signal amplitude at baseline and post intervention, for both control and treatment groups. Signal quality index (SQI) values, calculated from the beats^18^, showed similar distributions across measurement phases and procedural groups for each sensor (Fig. 8). Exemplary waveforms for ECG, PPG, and IPG from one participant, aggregated from 60 beats for each measurement phase and procedural group, with labeled SQI values, are shown in Fig. 9.

**Fig. 10:**
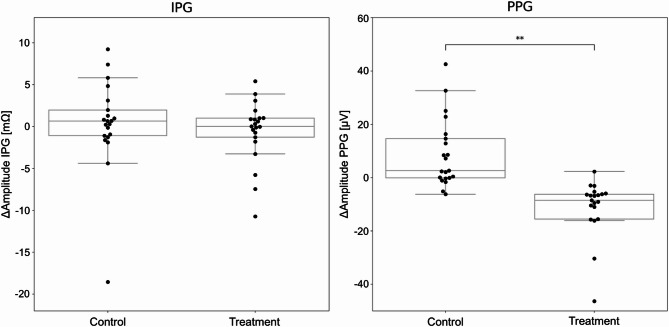
Changes in IPG and PPG amplitudes (post intervention - baseline) across control and treatment groups. ** marks a statistically significant change of *p* < 0.01.

**Fig. 11:**
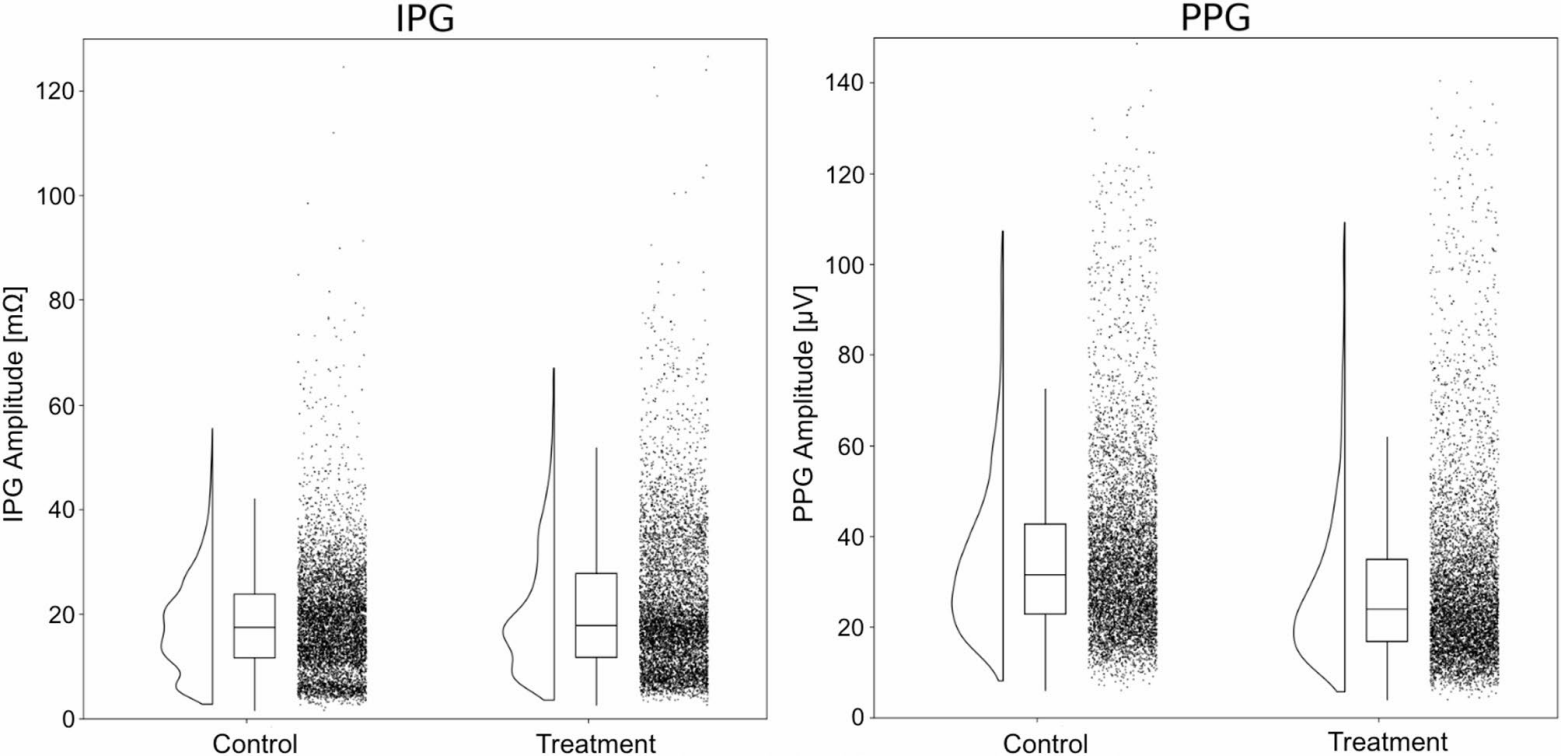
Distributions of the amplitudes from IPG (left) and PPG (right) sensors. Distributions represent all beats from the 12 min-long protocol for the control and treatment groups respectively.

A paired t-test revealed a statistically significant difference in PPG signal amplitude between the treatment and control groups (p_adj_ = 0.004) (Fig. 10; Table 3). The treatment group exhibited a mean decrease of 14.3 µV (a 41% reduction) in PPG amplitude, whereas the control group showed a mean increase of 8.7 µV (a 27% increase). In contrast, no statistically significant difference was observed in the change in IPG amplitude between the two groups (p_adj_ = 1.0) (Fig. 11; Table 3).

To investigate the potential interaction between the procedural group (control, treatment) and measurement phase (baseline, post-intervention), two-way repeated measures ANOVAs were performed for each physiological measure. For PPG amplitude, a significant (procedural group) x (measurement phase) interaction was observed, F(1, 20) = 14.2, *p* = 0.001, η_g_^2^​ = 0.094. This finding is consistent with the significant difference in amplitude changes between the two groups, as previously indicated by the paired t-test. For IPG amplitude, the (procedural group) x (measurement phase) interaction was not statistically significant, F(1, 20) = 0.38, *p* = 0.55, η_g_^2^​ ​= 0.0005. This indicates that the pattern of change in IPG amplitude from baseline to post intervention was similar across the control and treatment procedures.

**Table 2:** Aggregated signal amplitude analysis of all participants across respective groups and measurement phases.

Group	Measurement Phase	PPG Amplitude (Mean, SD), µV	IPG Amplitude (Mean, SD), mΩ
Control	Baseline	32.6, 14.9	18.8, 10.9
	Post Intervention	41.3, 25.3	19.1, 12.6
Treatment	Baseline	34.9, 24.1	22.4, 12.7
	Post Intervention	20.6, 10.2	21.8, 13.2

**Table 3:** Paired t-test results for sensor amplitude metrics. Statistically-significant differences are indicated as follows: ns (not significant) for p_adj_ > 0.05, * for p_adj_ < 0.05, ** for p_adj_ < 0.01, and *** for p_adj_ < 0.001).

Sensor	Metric	Group	Test type	t-stats	*p*-value	Adj. *p*-value	Stat. Sig.
IPG_Z	Amplitude	control	baseline vs. post intervention	−0.29	0.77	1.0	ns
IPG_Z	Amplitude	treatment	baseline vs. post intervention	0.75	0.46	1.0	ns
IPG_Z	ΔAmplitude	n/a	control vs. treatment	0.61	0.55	1.0	ns
PPG	Amplitude	control	baseline vs. post intervention	−3.1	0.006	0.02	*
PPG	Amplitude	treatment	baseline vs. post intervention	3.6	0.002	0.006	**
PPG	ΔAmplitude	n/a	control vs. treatment	3.8	0.001	0.004	**

### Timing analysis

The same peak finding approach described in Sect. “Signal amplitude analysis” was employed for timing analysis. Two timing metrics were investigated to see if there were any meaningful differences between procedural groups or had relationships with measured blood pressure. Specifically, pulse arrival time (PAT) - a non-invasive proxy of arterial stiffness^8^ - was calculated for both IPG and PPG as the difference in time between the ECG R peak and the respective signals’ valley/foot. Additionally, the time difference between the valley of the IPG Z and the valley of PPG was calculated (ΔT_xPG_)^17^.

**Fig. 12:**
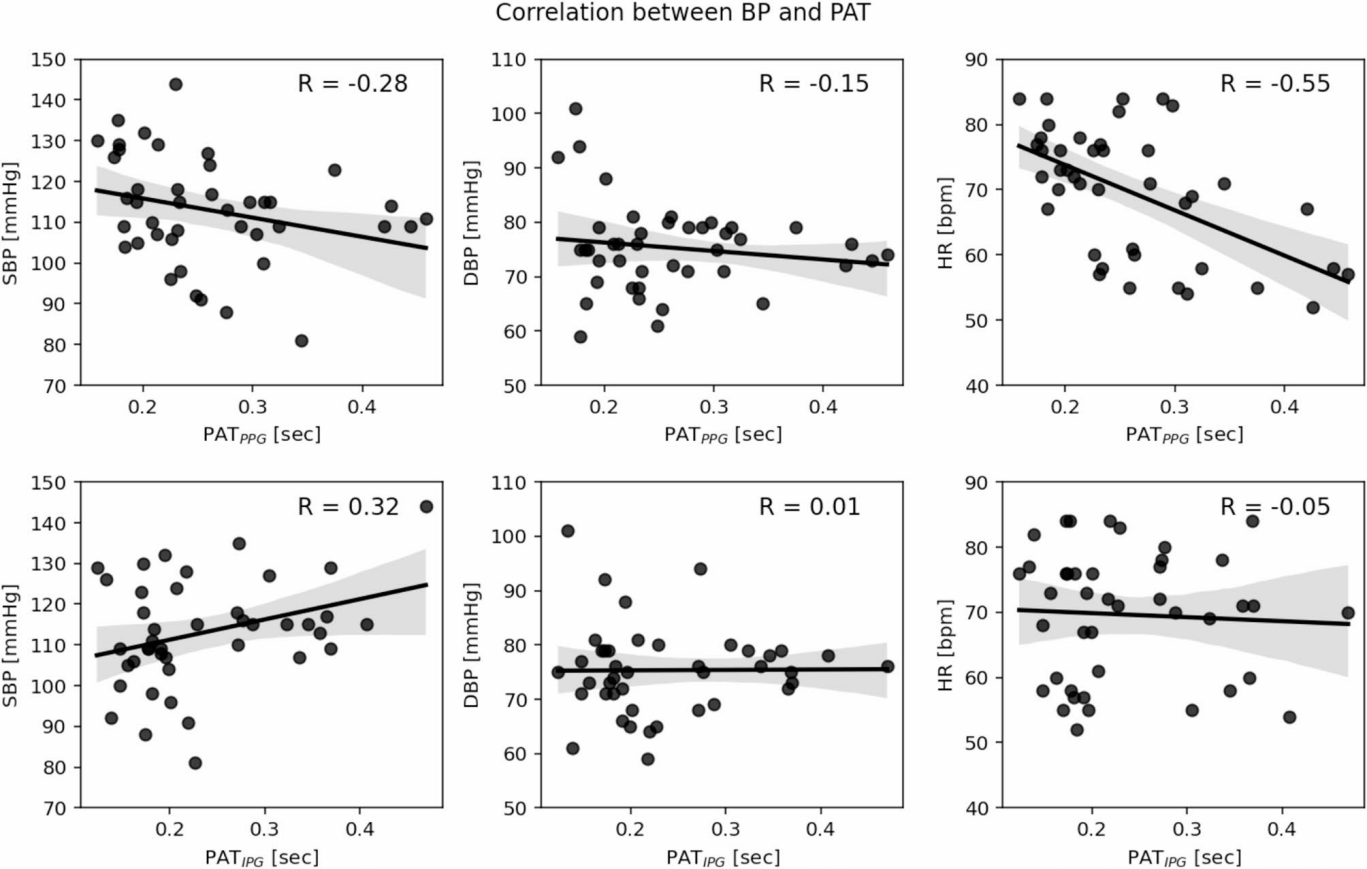
Correlation between Omron arm cuff measurements (SBP: left, DBP: middle, HR: right) and PAT (PPG-based: top, IPG-based: bottom). Pearson correlation coefficients (R) are marked.

**Fig. 13:**
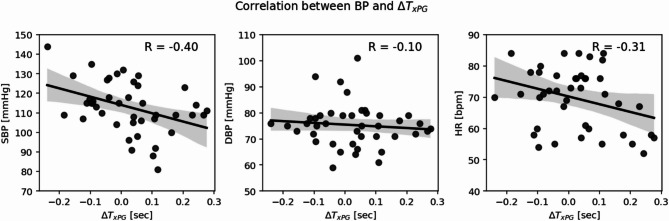
Correlation between Omron arm cuff measurements (SBP: left, DBP: middle, HR: right) and ΔT_xPG_. Pearson correlation coefficients (R) are marked.

Heart rate had a moderate correlation (Pearson correlation coefficient, *R* = −0.55) with PPG-derived PAT (Fig. 12). No significant correlations were observed between BP and PAT. A paired t-test revealed no statistically-significant differences in timing metrics between the control and treatment groups, or across the baseline and post intervention measurement phases (Table 6, Appendix). As shown in Fig. 13, ΔT_xPG_ had weak correlations with systolic blood pressure (SBP) (*R* = −0.40) and heart rate (*R* = −0.31). Pulse wave velocity (PWV) was also evaluated by dividing each subject’s height by their respective PAT. The PPG-derived PAT based PWV and SBP had a correlation coefficient of 0.41. For other PAT converted PWVs, there wasn’t a meaningful change in correlation coefficients.

## Discussion

This study investigated the differential responses of IPG and PPG to temperature-mediated peripheral vasoconstriction, providing valuable insights into their distinct sensitivities to superficial blood flow dynamics. The within-subject experimental design, incorporating a controlled localized cooling intervention, facilitated a direct and reliable comparison between the two sensing modalities. Crucially, the use of both real and fake ice cubes in the treatment and control groups, respectively, isolated the impact of temperature variation while mitigating potential confounding factors related to physical sensor disturbance.

Consistent with its known high sensitivity to superficial blood flow, the PPG signal exhibited a statistically significant reduction in amplitude following localized cooling, aligning with established literature on microvascular alterations^16^. This observed amplitude decrease is well-explained by several prominent PPG models. In the volumetric model, it is proposed that vasoconstriction directly reduces total blood volume within the illuminated tissue, leading to less light absorption and a diminished PPG signal^16^. Furthermore, constricted vessels have a reduced capacity for pulsatile expansion, contributing to a smaller signal. Similarly, the capillary mechanical movements model suggests that vasoconstriction diminishes the pressure pulse reaching capillaries, thereby reducing their pulsatile expansion and contraction, resulting in weaker signals. Recent research utilizing high-speed PPG imaging has visualized this phenomenon^9,10^. While the red blood cell orientation and deformation model might imply secondary effects due to vessel constriction, the primary impact of vasoconstriction is a direct reduction in blood volume and flow^12^. Overall, the observed cold-induced PPG amplitude drop appears to align well with both the volumetric and capillary mechanical movements models, with the red blood cell orientation and deformation model explanation being a secondary consideration.

Independent of these three physiological models for PPG - all of which consistently point towards superficial factors - the key finding that the PPG signal diminishes while the IPG signal remains stable challenges a purely superficial model for IPG’s origin. This observed stability of IPG amplitude during vasoconstriction necessitates a critical examination of its underlying physiological mechanisms and signal penetration depth. Specifically, if superficial blood volume, flow, and/or mechanical factors near the skin surface impact the PPG signal, a similar impact on IPG would be expected if its signal originated superficially; however, this impact was not observed. This leads to the hypothesis that IPG primarily senses deeper tissue, a notion supported by simulation-based measurements of radial artery bioimpedance by Petsi et al. (2021)^19^. Their evaluation of a human forearm 3D-model, created in Comsol Multiphysics with inline Ag/AgCl electrodes placed on a radial artery (similar to our setup), revealed that injected current from the outer electrodes flowed directly through the radial artery with the inner electrodes sensing the resulting voltage potential. The human forearm model was created using MRI-scan-based geometry and known conductivity distributions for skin, fat, bone, muscle, and tissue.

When investigating timing metrics, no significant variations in PAT were observed for either IPG or PPG following localized cooling. PAT is sometimes used as a proxy for arterial stiffness and blood pressure fluctuations^8^. The absence of significant PAT changes suggests that the localized cooling may not have induced substantial changes in arterial stiffness or systematic blood pressure within the observed timeframe, which is consistent with the stable blood pressure measurements obtained via oscillometry. PPG-derived PAT had a moderate correlation with HR, which has also been observed across several studies^20,21^, a consequence of the cardiac cycle timing. A statistically-significant decrease in heart rate was also observed, likely due to participants sitting still for a relatively long period of time and asking them to relax. These findings suggest that the localized vasoconstriction primarily affected the microvasculature, with minimal impact on larger arterial compliance and overall systemic hemodynamics.

Crucially, the cold intervention ensured a homogenous temperature decrease across sensing sites. Skin temperature measurements at proximal, central, and distal sensor locations all showed a statistically significant decrease during the cooling intervention, confirming the consistent application of the thermal stimulus. This uniformity, combined with both PPG and IPG sensors being placed directly over the radial artery region, supports the validity of direct comparisons between the two modalities. Separately, the observed increase in PPG amplitude in the control group is likely attributable to localized vasodilation, possibly due to the insulating and a mild heating effect of the towel^22^.

There are several limitations to this study. Firstly, the participant cohort consists primarily of Google employees located in the San Francisco Bay Area, potentially limiting the generalization of the findings to the broader population. Future studies should aim for greater demographic diversity, including diverse skin tones, to enhance robustness of the conclusions. This includes further exploration of the weak to moderate correlation observed in this study between PAT and SBP, wherein larger and more diverse cohorts might show more granular insights into this relationship. Secondly, further investigations into the signal penetration of PPG and IPG in these interventional scenarios with smaller form factors seems a natural extension of this work. Additionally, PPG and IPG sensors could be further optimized to better accommodate wearable form factors. For instance, multi-path PPG with varying source-detector distances could be considered in future studies instead of single channel PPG to account for penetration depth dependency. Electrode design parameters for IPG might also be further optimized such as its positioning and product-friendly material choice. While the excitation frequency selected for this study (100 kHz) is typically chosen for IPG, future research could explore IPG signals at higher or lower frequencies to understand their implications for sensitivity or the impact of confounders, particularly relevant for dry electrode IPG.

## Conclusion

This study investigated the impact of reduced superficial blood flow on IPG and PPG signals. The findings suggest that IPG signals, unlike PPG signals, remain relatively stable despite significant changes in superficial blood flow due to localized cold-induced vasoconstriction. This observation has implications for the potential use of IPG in wearable devices for monitoring deeper blood flow and cardiovascular activity wherein IPG can provide a valuable complementary measure to PPG. This information can be used to develop more robust and accurate models related to blood flow, paving the way for more personalized and adaptive human-computer interactions.

## Supplementary Information

Below is the link to the electronic supplementary material.

Supplementary Material 1

## Data Availability

The datasets generated during and/or analysed during the current study are available from the corresponding authors on reasonable request.
