# Supplementary material for: Differential sensitivity of impedance plethysmography and photoplethysmography sensors to temperature-induced peripheral vasoconstriction
Source: Sci Rep. 2026 Jan 31;16:6828. doi: 10.1038/s41598-026-36563-6 (PMC12916965; doi:10.1038/s41598-026-36563-6)
Supplement: Supplementary file 1 — Supplementary Material 1 [file 41598_2026_36563_MOESM1_ESM.docx]

# Appendix

**Table 4:** Paired t-test results for skin temperature. Statistically-significant differences are indicated as follows: ns (not significant) for p_adj_ > 0.05, * for p_adj_ < 0.05, ** for p_adj_ < 0.01, and *** for p_adj_ < 0.001).

| **Sensor** | **Metric** | **Group** | **Test Type** | **t-stats** | **p-value** | **Adj. p-value** | **Stat. Sig.** |
| --- | --- | --- | --- | --- | --- | --- | --- |
| IR thermal camera | Temp_proximal_ | control | baseline vs. post intervention | -2.7 | 0.01 | 0.04 | * |
| IR thermal camera | Temp_proximal_ | treatment | baseline vs. post intervention | 12 | 0.00 | 0.00 | *** |
| IR thermal camera | ΔTemp_proximal_ | n/a | control vs. treatment | 12 | 0.00 | 0.00 | *** |
| IR thermal camera | Temp_central_ | control | baseline vs. post intervention | -3.4 | 0.003 | 0.009 | ** |
| IR thermal camera | Temp_central_ | treatment | baseline vs. post intervention | 14.2 | 0.00 | 0.00 | *** |
| IR thermal camera | ΔTemp_central_ | n/a | control vs. treatment | 14 | 0.00 | 0.00 | *** |
| IR thermal camera | Temp_distal_ | control | baseline vs. post intervention | -2.4 | 0.03 | 0.08 | ns |
| IR thermal camera | Temp_distal_ | treatment | baseline vs. post intervention | 19 | 0.00 | 0.00 | *** |
| IR thermal camera | ΔTemp_distal_ | n/a | control vs. treatment | 18 | 0.00 | 0.00 | *** |

**Table 5:** Paired t-test results for blood pressure and heart rate. Statistically-significant differences are indicated as follows: ns (not significant) for p > 0.05, * for p < 0.05, ** for p < 0.01, and *** for p < 0.001).

| **Sensor** | **Metric** | **Test Type** | **t-stats** | **p-value** | **Stat. Significance** |
| --- | --- | --- | --- | --- | --- |
| Arm cuff | SBP | start vs. end | 0.76 | 0.46 | ns |
| Arm cuff | DBP | start vs. end | 0.0 | 1.0 | ns |
| Arm cuff | HR | start vs. end | 2.3 | 0.03 | * |

**Table 6:** Paired t-test results for sensor timing metrics. Statistically-significant differences are indicated as follows: ns (not significant) for p_adj_ > 0.05, * for p_adj_ < 0.05, ** for p_adj_ < 0.01, and *** for p_adj_ < 0.001).

| **Sensor** | **Metric** | **Group** | **Test Type** | **t-stats** | **p-value** | **Adj. p-value** | **Stat. Sig.** |
| --- | --- | --- | --- | --- | --- | --- | --- |
| IPG_Z & ECG | PAT | control | baseline vs. post intervention | 1.5 | 0.16 | 0.48 | ns |
| IPG_Z & ECG | PAT | treatment | baseline vs. post intervention | -0.08 | 0.93 | 1.0 | ns |
| IPG_Z & ECG | ΔPAT | n/a | control vs. treatment | -1.3 | 0.22 | 0.66 | ns |
| PPG & ECG | PAT | control | baseline vs. post intervention | 0.60 | 0.56 | 1.0 | ns |
| PPG & ECG | PAT | treatment | baseline vs. post intervention | -1.8 | 0.08 | 0.24 | ns |
| PPG & ECG | ΔPAT | n/a | control vs. treatment | -1.6 | 0.12 | 0.36 | ns |
| IPG_Z & PPG | ΔT_xPG_ | control | baseline vs. post intervention | -0.98 | 0.34 | 1.0 | ns |
| IPG_Z & PPG | ΔT_xPG_ | treatment | baseline vs. post intervention | -1.4 | 0.19 | 0.57 | ns |
| IPG_Z & PPG | Δ(ΔT_xPG_) | n/a | control vs. treatment | -0.37 | 0.72 | 1.0 | ns |
